# Twelve-Month BMI and Metabolic Changes After Roux-en-Y Gastric Bypass in Women with Severe Obesity

**DOI:** 10.3390/jcm15155764

**Published:** 2026-07-23

**Authors:** Augusto Cardoso Sgarioni, Giovani Schulte Farina, Tulio Slongo Bressan, Milena Prigol Dalfovo, Gabriel Michelin De Carli, Henrique João Prataviera Giovanardi, Guilherme Schumacher Giovanardi, Luciano da Silva Selistre, Rosa Maria Rahmi Garcia

**Affiliations:** 1 Graduate Program in Health Sciences, University of Caxias do Sul, Caxias do Sul 95070-560, Brazil; 2 Department of Surgery, General Hospital of Caxias do Sul, Caxias do Sul 95070-561, Brazil; 3 School of Medicine, University of Caxias do Sul, Caxias do Sul 95070-560, Brazil

**Keywords:** Roux-en-Y gastric bypass, bariatric surgery, severe obesity, body mass index, women, metabolic outcomes

## Abstract

**Background/Objectives:** Women-specific short-term body mass index and metabolic changes after Roux-en-Y gastric bypass remain incompletely characterized. This retrospective cohort study evaluated 12-month changes in body mass index, glycemic markers, and lipid profile after Roux-en-Y gastric bypass in women with severe obesity treated at a public bariatric reference center in Brazil. **Methods:** Women who underwent Roux-en-Y gastric bypass between September 2017 and January 2020 were assessed preoperatively and at 30 days, 3 months, 6 months, and 12 months postoperatively. Longitudinal changes were analyzed using linear mixed-effects models. **Results:** Of 168 women who underwent bariatric surgery, 150 had complete 12-month follow-up data. Mean age was 43.6 ± 9.8 years and mean preoperative body mass index was 45.4 ± 5.4 kg/m^2^. Body mass index decreased progressively from 45.5 kg/m^2^ at baseline to 31.5 kg/m^2^ at 12 months. Fasting plasma glucose and HbA1c declined significantly within the first postoperative month and remained improved. Total cholesterol decreased at 30 days, whereas low-density lipoprotein (LDL) cholesterol and triglycerides decreased by 6 months. Among baseline users, 86% discontinued antihyperglycemic therapy, 87% lipid-lowering therapy, and 72% antihypertensive therapy; these exploratory medication-discontinuation outcomes should not be interpreted as validated remission of diabetes, dyslipidemia, or hypertension. **Conclusions:** Roux-en-Y gastric bypass was associated with substantial short-term body mass index reduction and improved cardiometabolic markers among women.

## 1. Introduction

Obesity is a major and rapidly growing global health problem associated with multiple cardiometabolic and systemic comorbidities, including type 2 diabetes mellitus (T2DM), dyslipidemia, cardiovascular disease, and several cancers, ultimately reducing quality of life and life expectancy [1]. Worldwide, obesity prevalence nearly tripled between 1975 and 2016, affecting more than 650 million adults; in Brazil, 57.5% of adults have excess body weight, and 21.5% have obesity [2,3].

Body mass index (BMI) remains the most widely used measure to define obesity, stratify disease severity, and guide eligibility for bariatric surgery [4,5]. Although BMI does not capture body composition, fat distribution, sex, age, or ethnicity, it remains a practical and standardized measure for assessing obesity severity and monitoring postoperative anthropometric change [6,7]. This is particularly relevant because many bariatric studies report percentage weight loss or excess weight loss, whereas longitudinal BMI trajectories provide a direct and clinically interpretable measure aligned with diagnostic classification and surgical indication criteria.

Lifestyle interventions, including dietary modification, physical activity, and behavioral strategies, are recommended as first-line therapy [5,8]. However, sustained weight loss remains difficult to achieve, with frequent weight regain even when combined with pharmacological treatment [9]. Bariatric and metabolic surgery produces greater and more durable weight loss and is associated with improvement or resolution of obesity-related comorbidities [10]. Roux-en-Y gastric bypass (RYGB), in particular, has been associated with substantial excess weight loss; improvements in diabetes, dyslipidemia, and hypertension [11]; and long-term reductions in all-cause mortality in population-based studies [12]. Optimal outcomes depend not only on the procedure itself, but also on appropriate patient selection, standardized surgical technique, and structured multidisciplinary follow-up [13].

Women are particularly relevant in bariatric surgery research. Although adult obesity prevalence is broadly similar between sexes, women account for around 80% of patients undergoing bariatric surgery [14,15]. This predominance likely reflects biological, reproductive, and psychosocial factors, as obesity has sex-specific consequences across the female life course, including effects on reproductive health, pregnancy outcomes, and long-term cardiometabolic and oncologic risk [16,17,18]. Women-only cohorts may therefore provide more specific insight into postoperative trajectories by reducing heterogeneity related to sex differences in obesity-related risk, treatment-seeking behavior, and metabolic response.

Despite extensive literature on bariatric surgery, most published studies include mixed-sex populations, compare different surgical techniques, or emphasize percentage-based weight-loss outcomes rather than longitudinal BMI change. Moreover, women-only studies after bariatric surgery have more commonly focused on long-term outcomes, leaving short-term trajectories of BMI and metabolic markers after RYGB less well characterized [19]. In contrast, the present study focuses on absolute BMI and metabolic trajectories during the first postoperative year in a women-only cohort undergoing a single standardized RYGB technique within a Brazilian public-sector reference program, a setting in which real-world outcome data remain particularly relevant given limited access to bariatric surgery in the public health system [19,20,21]. Therefore, this study aimed to evaluate 12-month longitudinal changes in BMI, glycemic markers, and lipid profile after open RYGB in women with severe obesity treated at a public bariatric reference center.

## 2. Materials and Methods

### 2.1. Study Design and Population

This was a retrospective cohort study including women who underwent bariatric and metabolic surgery between September 2017 and January 2020. It was conducted at the General Hospital of Caxias do Sul (GHCS) and the University of Caxias do Sul (UCS), Brazil, which constitute the 5th Regional Health Coordination Reference Service for Bariatric and Metabolic Surgery of Rio Grande do Sul.

All eligible patients who underwent primary bariatric surgery during the study period were considered for inclusion. Eligibility followed Brazilian Ministry of Health criteria (Ordinance No. 492), including severe obesity, defined as BMI ≥ 40 kg/m^2^ or BMI ≥ 35 kg/m^2^ with obesity-related comorbidities, after failure of conservative treatment. Additional criteria included age 18–65 years, absence of obesity secondary to endocrine disorders, adequate cognitive capacity and social support, commitment to long-term postoperative follow-up, and absence of active substance abuse, severe psychiatric disorders, or recent suicide attempts.

For this study, we included women aged ≥18 years who underwent open RYGB and had complete outpatient follow-up data for 12 months after surgery. Patients were excluded if they underwent bariatric procedures other than RYGB, revisional surgery, had missing or incomplete clinical or laboratory data, or became pregnant during follow-up. The study followed the Declaration of Helsinki and Brazilian National Health Council Resolution No. 466/2012 and was approved by the Research Ethics Committee of the UCS (approval number 39222020.5.0000.5341).

### 2.2. Surgical Procedure and Follow-Up

All participants underwent RYGB via laparotomy, performed by the same surgical team. This approach reflected the institutional surgical practice and availability of the public reference service during the study period, when laparoscopic RYGB was not routinely performed at the center. The procedure included the creation of an approximately 50 mL gastric pouch and Roux-en-Y reconstruction with 100 cm alimentary and biliopancreatic limbs.

Preoperative care followed a structured multidisciplinary program lasting approximately 4–6 months, including bariatric surgery, medical, nutritional, psychological, and nursing evaluations, as well as educational sessions focused on surgical preparation, nutritional adaptation, and long-term lifestyle changes. Preoperative weight-loss targets were individualized according to baseline BMI.

Postoperatively, participants were followed for 12 months according to a standardized multidisciplinary protocol involving bariatric surgeons, clinicians, nutritionists, psychologists, nurses, and other specialists as needed. All participants received structured nutritional counseling, behavioral support, medical follow-up, and uniform dietary and lifestyle guidance throughout follow-up.

### 2.3. Data Collection and Outcomes

Clinical and laboratory data were obtained from the institutional bariatric surgery database and supplemented by electronic medical records when necessary. Data were collected at five time points: preoperative assessment closest to surgery (T1), 30 days (T2), 3 months (T3), 6 months (T4), and 12 months (T5) postoperatively.

Collected variables included age, ethnicity, smoking status, place of residence, comorbidities (T2DM, arterial hypertension, and dyslipidemia), anthropometric measures (height, weight, and BMI), metabolic markers (fasting plasma glucose, glycated hemoglobin [HbA1c], total cholesterol, low-density lipoprotein [LDL]-cholesterol, high-density lipoprotein [HDL]-cholesterol, and triglycerides), and use of antihyperglycemic, antihypertensive, and lipid-lowering medications at all time points.

The primary anthropometric outcome was longitudinal absolute BMI. Secondary metabolic outcomes were fasting plasma glucose, HbA1c, total cholesterol, LDL-cholesterol, HDL-cholesterol, and triglycerides. Medication use was ascertained at each follow-up visit by the surgical team through patient report and review of recorded prescriptions for daily medications. Medication discontinuation was calculated descriptively among participants using the corresponding medication class at baseline and was defined as the absence of recorded use of that medication class at the 12-month visit. This measure was interpreted as medication de-escalation rather than comorbidity remission, because standardized remission criteria were not applied.

### 2.4. Statistical Analysis

Continuous variables were summarized as mean ± standard deviation or median with interquartile range, according to the distribution assessed by the Shapiro–Francia test. Categorical variables were described as counts and percentages. Because the primary analysis required a complete 12-month follow-up, analyses were performed using a complete-case cohort.

Longitudinal changes in BMI and metabolic outcomes were analyzed using linear mixed-effects models, with time as a fixed effect and participant-specific random intercepts to account for within-subject correlation. Models were estimated using maximum likelihood and applied consistently to BMI, fasting glucose, HbA1c, and lipid parameters.

When a significant overall time effect was identified, pairwise comparisons between time points were performed using Tukey-adjusted post hoc contrasts. Post hoc contrasts included both baseline-referenced comparisons, used to identify postoperative time points that differed from the preoperative value, and consecutive time point comparisons, used to evaluate interval-to-interval changes. Temporal trends were graphically displayed using third-degree polynomial regression curves to accommodate heterogeneity in follow-up timing. Ninety-five percent confidence intervals were obtained using bias-corrected and accelerated bootstrap resampling with 2000 participant-level iterations to preserve the repeated-measures structure. Two-sided *p*-values were adjusted for multiple testing using the Holm–Bonferroni procedure, with statistical significance set at *p* < 0.05. Analyses were performed in R software version 4.1.1 using the nlme version 3.1-170, tidyr version 1.3.2, and ggplot2 version 4.0.3 packages.

## 3. Results

### 3.1. Study Population

**Figure 1:**
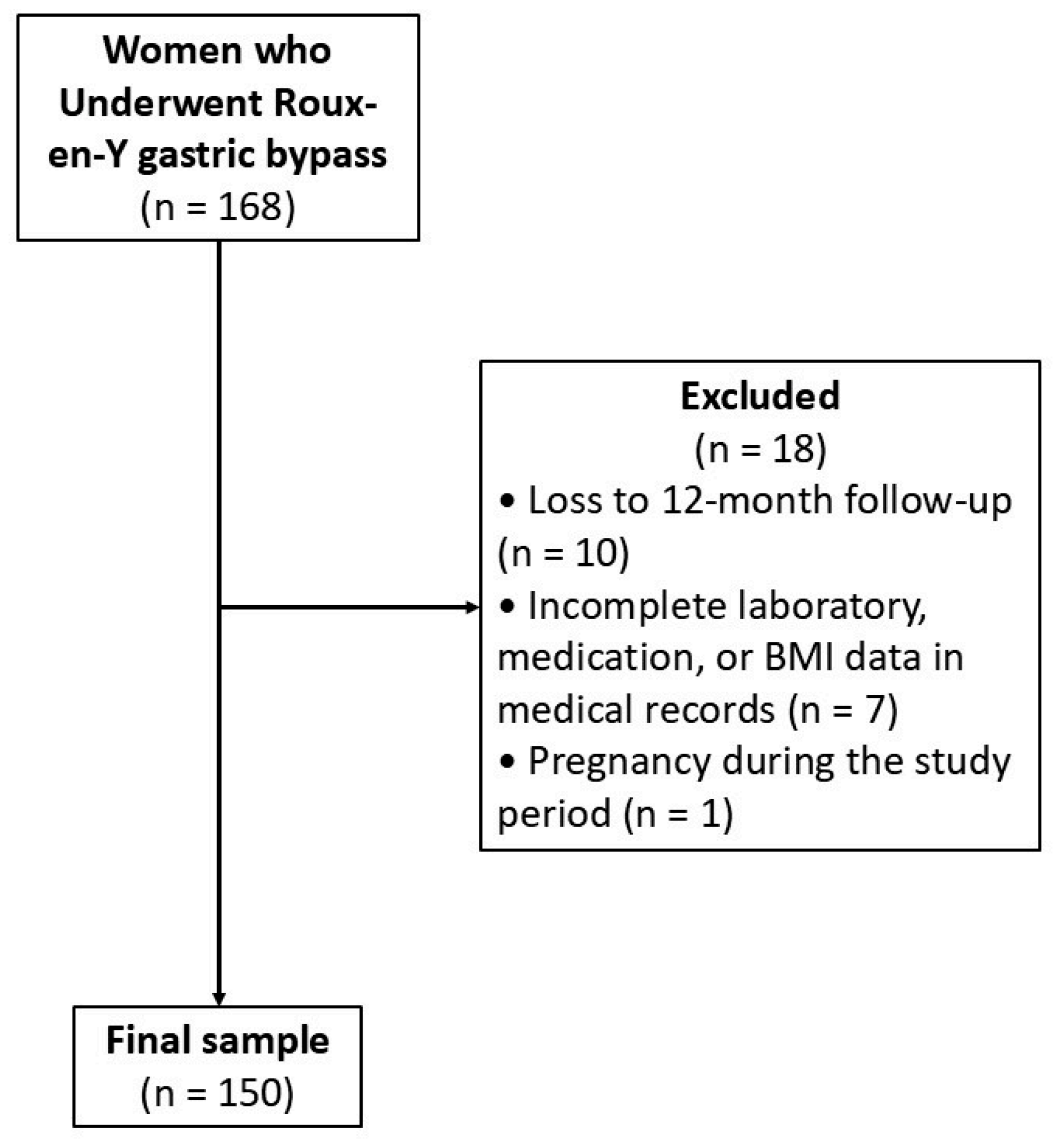
Study flowchart. Flow diagram showing patient selection for the final analytical cohort. Between September 2017 and January 2020, 168 individuals underwent bariatric surgery at the study center. Of these, 12 were excluded for being male and 18 because of loss to clinical follow-up, incomplete laboratory or BMI data, or pregnancy during the study period. The final study sample included 150 women who underwent open Roux-en-Y gastric bypass and had complete 12-month follow-up data.

Between September 2017 and January 2020, 168 women underwent bariatric surgery at the study center. Of these, 18 were excluded because of loss to 12-month follow-up (n = 10), incomplete laboratory or BMI data (n = 7), or pregnancy during follow-up (n = 1), resulting in an attrition rate of 10.8%. The final analytical sample included 150 women with complete 12-month follow-up data. No deaths occurred during follow-up (Figure 1).

Baseline demographic, clinical, and laboratory characteristics are summarized in Table 1. Mean age was 43.6 ± 9.8 years, and mean preoperative BMI was 45.4 ± 5.4 kg/m^2^. At baseline, 57 participants (38.0%) had T2DM, 119 (79.3%) had arterial hypertension, and 46 (30.7%) had dyslipidemia.

### 3.2. Longitudinal Changes in Body Mass Index

**Figure 2:**
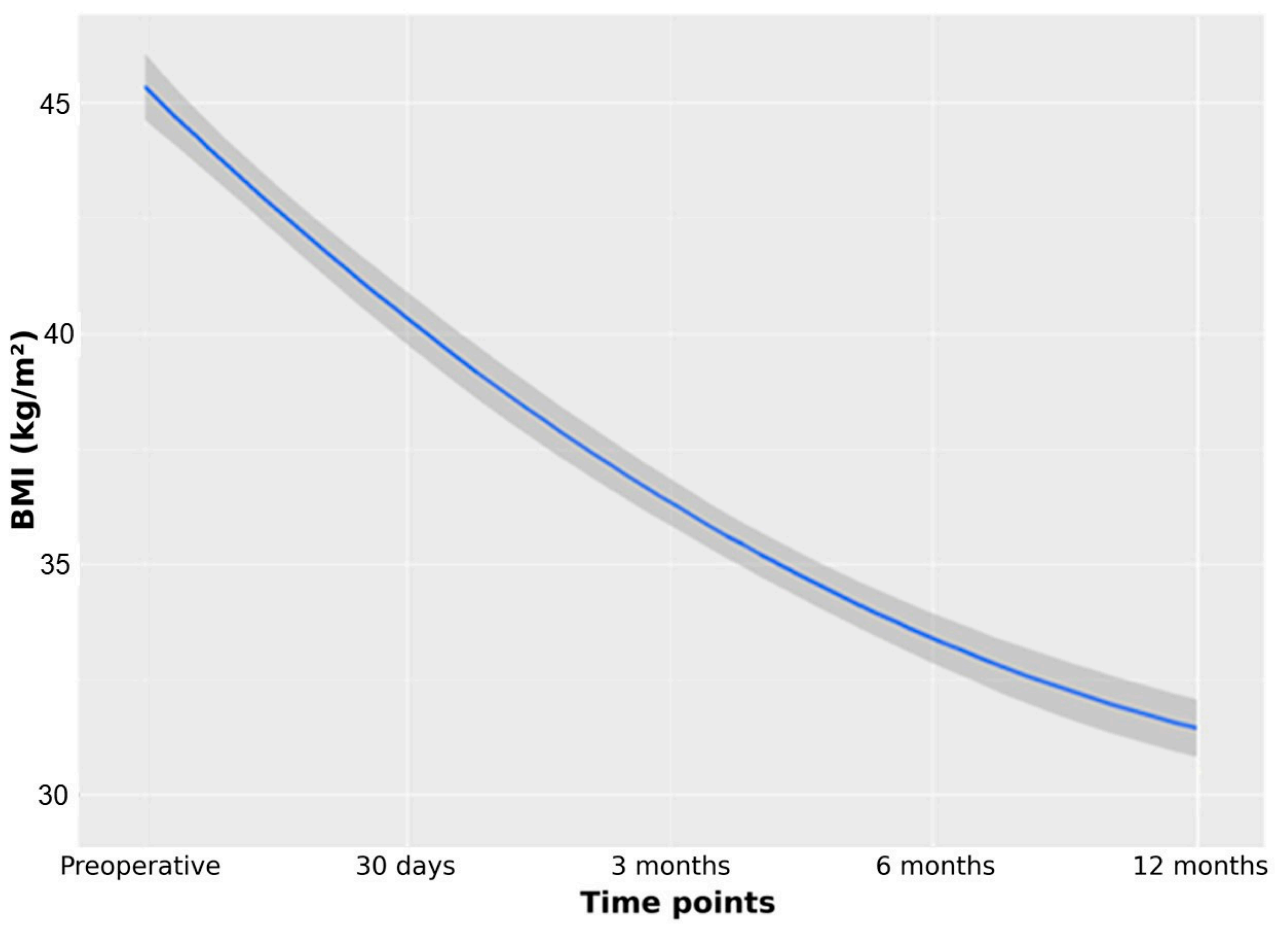
Longitudinal trajectory of body mass index during the 12-month follow-up. Representative curve showing the change in body mass index (BMI) from the preoperative assessment to 30 days, 3 months, 6 months, and 12 months after open Roux-en-Y gastric bypass. BMI decreased progressively throughout follow-up, with the greatest absolute reduction occurring during the early postoperative period and continued decline observed up to 12 months.

BMI decreased progressively and significantly throughout the 12-month follow-up period (Table 2 and Figure 2). Mean BMI declined from 45.5 kg/m^2^ at baseline to 40.0 kg/m^2^ at 30 days, 36.5 kg/m^2^ at 3 months, 33.1 kg/m^2^ at 6 months, and 31.5 kg/m^2^ at 12 months. Reductions were significant between every consecutive time point, indicating continuous weight loss throughout follow-up.

The largest reduction occurred between baseline and 30 days, with a mean decrease of 5.5 kg/m^2^ (95% CI, −6.0 to −4.5; *p* < 0.001). Subsequent reductions remained significant but became progressively smaller: −3.5 kg/m^2^ from 30 days to 3 months, −3.5 kg/m^2^ from 3 to 6 months, and −1.5 kg/m^2^ from 6 to 12 months. Overall, BMI decreased by approximately 14.0 kg/m^2^ over 12 months.

### 3.3. Longitudinal Changes in Fasting Plasma Glucose

**Figure 3:**
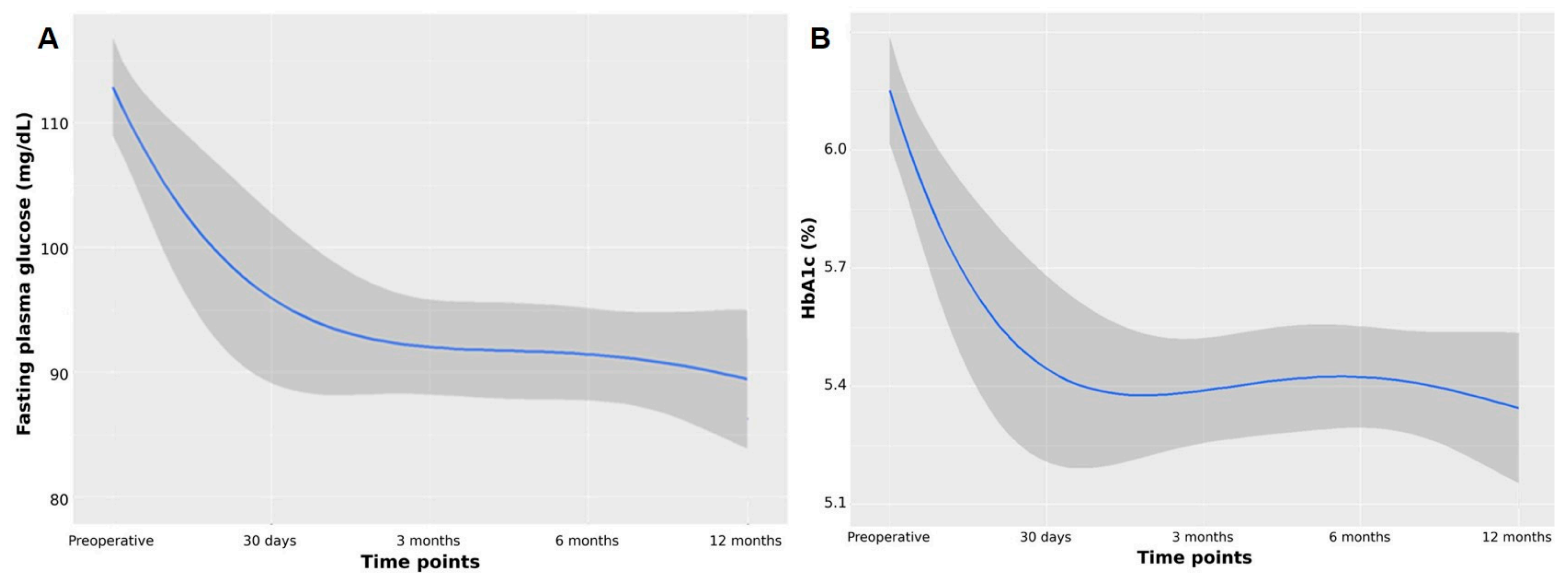
Longitudinal trajectories of glycemic markers during the 12-month follow-up. Representative curves showing changes in fasting plasma glucose (**A**) and glycated hemoglobin, HbA1c (**B**) from the preoperative assessment to 30 days, 3 months, 6 months, and 12 months after open Roux-en-Y gastric bypass. Both glycemic markers decreased markedly during the first postoperative month and remained at lower levels throughout follow-up, indicating early improvement in glycemic control that was maintained through 12 months.

Fasting plasma glucose showed an early postoperative decline followed by stabilization (Table 2 and Figure 3A). The main reduction occurred between baseline and 30 days, with a mean decrease of 20.5 mg/dL (95% CI, −31.0 to −9.5; *p* < 0.001). Mean fasting glucose was approximately 93.0 mg/dL at 30 days and remained stable thereafter, with values around 90–93 mg/dL from 3 to 12 months.

No significant differences were observed between consecutive postoperative time points after the first month. However, fasting glucose remained significantly lower than baseline at 3, 6, and 12 months, indicating maintenance of the early glycemic improvement throughout follow-up.

### 3.4. Longitudinal Changes in Glycated Hemoglobin (HbA1c)

HbA1c followed a similar pattern, with an early decline followed by stabilization through 12 months (Table 2 and Figure 3B). Mean HbA1c decreased from 6.10% at baseline to 5.40% at 30 days, corresponding to a mean reduction of 0.76 percentage points (95% CI, −1.09 to −0.43; *p* < 0.001). HbA1c then remained stable, with mean values of 5.41% at 3 months, 5.40% at 6 months, and 5.36% at 12 months.

There were no significant differences between consecutive postoperative time points from 30 days onward. Nevertheless, HbA1c remained significantly lower than baseline at 3, 6, and 12 months, confirming improvement in glycemic control that was maintained through 12 months.

### 3.5. Longitudinal Changes in Lipid Profile

The lipid profile showed a more heterogeneous pattern than the glycemic markers, with early improvement in total cholesterol, delayed reductions in LDL-cholesterol and triglycerides, and a nonsignificant upward trend in HDL-cholesterol.

**Figure 4:**
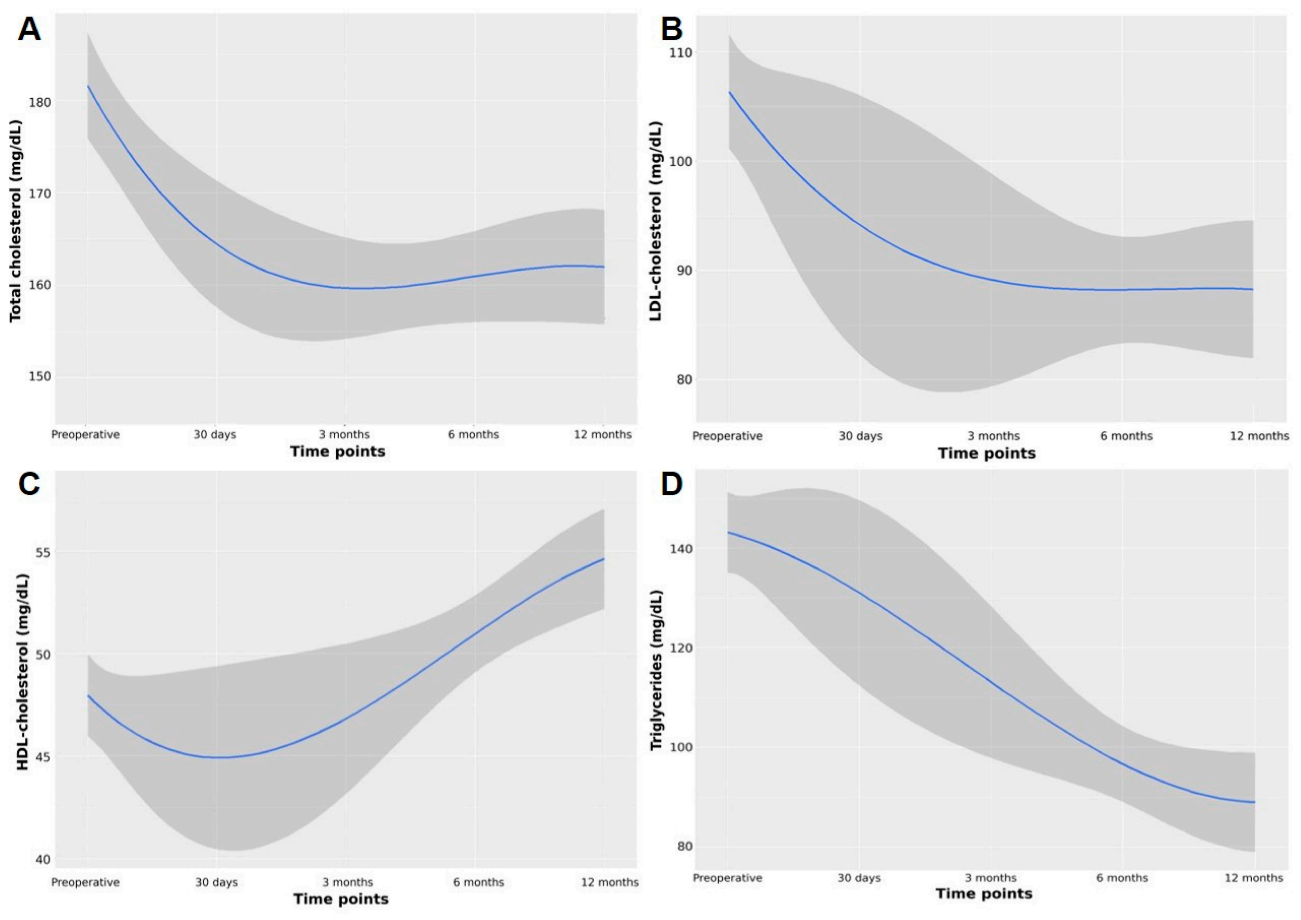
Longitudinal trajectories of lipid markers during the 12-month follow-up. Representative curves showing changes in total cholesterol (**A**), low-density lipoprotein (LDL)-cholesterol (**B**), high-density lipoprotein (HDL)-cholesterol (**C**), and triglycerides (**D**) from the preoperative assessment to 30 days, 3 months, 6 months, and 12 months after open Roux-en-Y gastric bypass. Total cholesterol decreased early after surgery and remained lower than baseline throughout follow-up. LDL-cholesterol showed a more gradual decline, with lower values observed in the later postoperative assessments. HDL-cholesterol showed a slight early decrease followed by a progressive increase, reaching its highest levels at 12 months. Triglyceride levels declined progressively over time, with lower values observed in the later postoperative assessments and the lowest levels at 12 months.

Total cholesterol decreased rapidly after surgery (Table 2 and Figure 4A), from 182.5 mg/dL at baseline to 163.0 mg/dL at 30 days, a significant reduction of 19.5 mg/dL (*p* < 0.001). It then remained relatively stable, with mean values of 161.5 mg/dL at both 3 and 6 months and 158.0 mg/dL at 12 months. Although no further significant reductions were observed between consecutive postoperative time points, total cholesterol remained significantly lower than baseline at 3, 6, and 12 months.

LDL-cholesterol showed a more gradual pattern of improvement (Table 2 and Figure 4B). Mean LDL-cholesterol decreased from 106.5 mg/dL at baseline to 81.5 mg/dL at 30 days, although this change was not significant in pairwise comparison. LDL-cholesterol was 88.0 mg/dL at 3 months, 89.5 mg/dL at 6 months, and 86.5 mg/dL at 12 months, with values significantly lower than baseline at 6 and 12 months.

HDL-cholesterol did not change significantly during follow-up (Table 2 and Figure 4C). Mean HDL-cholesterol was 48.0 mg/dL at baseline, fluctuated during the early postoperative period, and reached 55.0 mg/dL at 12 months. Although this pattern suggested a late upward trend, particularly after 6 months, neither consecutive nor baseline-to-12-month comparisons reached statistical significance.

### 3.6. Longitudinal Changes in Triglycerides

Triglyceride levels decreased progressively over 12 months, with significant reductions becoming evident from 6 months onward (Table 2 and Figure 4D). Mean triglycerides declined from 143.5 mg/dL at baseline to 116.5 mg/dL at 30 days and 118.0 mg/dL at 3 months. A more pronounced decrease was observed by 6 months, when mean triglycerides reached 95.5 mg/dL, and this improvement persisted at 12 months, with a mean value of 90.5 mg/dL.

Although consecutive time point comparisons were not statistically significant, triglyceride levels were significantly lower than baseline at both 6 and 12 months, suggesting gradual improvement in triglyceride metabolism that was maintained through 12 months.

### 3.7. Medication Use at 12 Months

At 12 months, a substantial proportion of participants had no recorded use of medications for obesity-related cardiometabolic comorbidities, based on patient reports and prescription reviews during follow-up visits. Among participants with the corresponding baseline condition or treatment indication, 86% were no longer receiving pharmacological treatment for T2DM, 87% were no longer receiving lipid-lowering therapy, and 72% were no longer receiving antihypertensive medication. These findings were considered exploratory and interpreted as pragmatic indicators of medication de-escalation after surgery, rather than formal remission of comorbidities.

## 4. Discussion

In this retrospective cohort of women with severe obesity undergoing RYGB, we observed substantial short-term improvements in BMI, glycemic markers, and lipid profile over 12 months. The temporal pattern was clinically informative: BMI declined progressively throughout follow-up, fasting plasma glucose and HbA1c improved early and then stabilized, and LDL-cholesterol and triglycerides showed more gradual reductions that became evident later. By restricting the analysis to women treated with a single surgical technique at a regional public reference center and followed by the same multidisciplinary team, this study provides a focused real-world assessment of short-term anthropometric and metabolic trajectories after RYGB in female patients [22,23,24].

The women-only design is an important feature of this study. Sex-related differences in metabolic outcomes after bariatric surgery have been reported, although findings are not entirely consistent. Kennedy-Dalby et al. [25] demonstrated comparable weight loss and metabolic improvements between men and women following bariatric procedures, whereas Lyon et al. [26], in more than 80,000 patients undergoing RYGB, reported higher baseline BMI and greater absolute weight loss in men. These findings suggest that sex may influence postoperative trajectories and support sex-specific analyses, particularly because women represent most patients undergoing bariatric surgery. Santini et al. [19] further illustrate the value of women-specific bariatric research, showing that post-menopausal women several years after RYGB had lower fat mass, lower visceral adipose tissue, preserved lean mass, and a better lipid profile than age- and BMI-matched non-operated controls. However, their long-term body composition focus differs from the present study, which characterized early BMI and metabolic trajectories during the first postoperative year. Because this study did not include a male comparison group, its findings should not be interpreted as evidence of a differential female response, but rather as a focused description of outcomes in a clinically important and highly represented population.

BMI decreased markedly and continuously over the 12-month follow-up period. Patients had already achieved substantial BMI reduction by 30 days postoperatively, and BMI continued to decline at each subsequent time point, culminating in a mean reduction of approximately 14 kg/m^2^ at 12 months. This trajectory supports the effectiveness of RYGB in producing clinically meaningful short-term anthropometric improvement. The magnitude of BMI reduction was consistent with major randomized and multicenter studies: the SLEEVEPASS trial reported an absolute BMI reduction of 14.8 kg/m^2^ at one year after RYGB [27], and the SM-BOSS trial reported 76.7% excess BMI loss at 12 months [28]. Shah et al. [29] also reported substantial 12-month BMI reduction after RYGB, although their study compared RYGB with adjustable gastric banding and focused on regional body composition changes assessed by three-dimensional photonic scanning. Unlike many studies that emphasize percentage weight loss or excess weight loss, the present analysis focused on BMI, a clinically standardized measure directly related to obesity classification and bariatric surgery eligibility criteria. The relevance of early postoperative trajectories is further supported by evidence showing that suboptimal weight loss occurs in approximately 9.2% of primary RYGB patients by 6 months, with female sex, higher baseline BMI, older age, and diabetes identified as predictors of suboptimal early response [30].

Improvement of T2DM and glycemic control is a central objective of contemporary bariatric and metabolic surgery, particularly given the global obesity and diabetes epidemics [31,32]. In the present cohort, 38% of participants had T2DM at baseline, although mean preoperative glycemic values were already within recommended targets [33]. Despite this relatively controlled baseline profile, fasting plasma glucose and HbA1c decreased significantly within the first postoperative month and remained improved throughout follow-up, suggesting early glycemic benefit followed by stabilization rather than progressive decline. Therefore, the observed glycemic improvements should be interpreted in the context of a cohort with relatively controlled baseline glycemic status and may not be directly generalizable to populations with poorly controlled T2DM, higher baseline HbA1c, greater medication burden, or more advanced beta-cell dysfunction. Additionally, at 12 months, 86% of participants were no longer using antihyperglycemic medications, providing an exploratory indicator of medication de-escalation rather than validated diabetes remission.

This early glycemic response is consistent with evidence showing that glycemic improvement after RYGB may occur within days, before substantial weight loss, allowing many patients to reduce or discontinue T2DM medications rapidly [34,35,36]. These effects are biologically plausible because glucose homeostasis after RYGB improves through weight loss-dependent and weight loss-independent mechanisms, including changes in nutrient delivery, insulin sensitivity, insulin secretion, incretin signaling, bile acid metabolism, and gut–liver–pancreas crosstalk [34]. Postoperative hormonal changes, including enhanced secretion of glucagon-like peptide-1 (GLP-1), peptide YY, and oxyntomodulin, may improve insulin secretion and sensitivity and contribute to rapid glycemic control [37], alongside reductions in insulin resistance driven by decreased adiposity and inflammatory signaling [36,38].

The different temporal patterns observed across metabolic outcomes likely reflect distinct underlying physiological mechanisms. Glycemic improvement after RYGB occurs rapidly and may precede substantial weight loss because of early changes in nutrient delivery, incretin secretion, and insulin sensitivity [39]. In contrast, improvements in LDL-cholesterol and triglycerides appear to depend more progressively on ongoing postoperative weight loss, changes in hepatic lipid metabolism, dietary adaptation, and reductions in insulin resistance, resulting in a more gradual response over time [40,41].

The present findings also align with clinical studies reporting high rates of T2DM improvement or remission after RYGB. Mingrone et al. [42] reported diabetes remission in 75% of patients two years after RYGB, with no remissions in the medically treated group. Other prospective studies have reported remission rates ranging from 58% to 90% at 12 months [43,44,45,46,47]. Longer-term observational data also support sustained glycemic benefit after bariatric surgery. Behrooznia et al. [48], in a predominantly female cohort of patients with diabetes followed for five years after bariatric surgery, reported 71% diabetes remission and significant reduction in oral antidiabetic medication use, although their study included multiple surgical techniques and was restricted to patients with diabetes. In the present study, the early decreases in fasting glucose and HbA1c are consistent with this evidence, although formal diabetes remission criteria were not applied.

The lipid profile showed a different temporal pattern. Obesity-related dyslipidemia is closely linked to insulin resistance and chronic low-grade inflammation [49]. In this cohort, total cholesterol decreased significantly within the first postoperative month and remained lower thereafter, whereas LDL-cholesterol and triglycerides improved more gradually, with significant reductions evident by 6 months and persisting through 12 months. HDL-cholesterol remained statistically stable, although a late upward trend was observed. Thus, while glycemic markers may respond rapidly after RYGB, lipid parameters may follow a more heterogeneous course, with atherogenic lipid reductions preceding or exceeding detectable HDL improvement within the first postoperative year.

These lipid findings align with previous studies reporting improved lipid profiles after RYGB. Nguyen et al. [50] observed reductions in total cholesterol, LDL-cholesterol, and triglycerides, along with increased HDL-cholesterol, and reported that 82% of patients discontinued lipid-lowering medications within one year. Similar temporal dynamics have been described in other cohorts, with early reductions in atherogenic lipids and delayed increases in HDL-cholesterol [51,52,53]. Recent MASLD-focused evidence also supports the broader metabolic benefits of bariatric surgery. Longsomboon et al. [10], in patients with biopsy-confirmed MASLD undergoing metabolic and bariatric surgery, reported significant postoperative improvements in HbA1c, LDL-cholesterol, and triglycerides, although their study included both sleeve gastrectomy and RYGB and was primarily designed to assess liver histological outcomes.

In the present study, 87% of participants were no longer receiving pharmacological treatment for dyslipidemia at 12 months, reinforcing the clinical relevance of the lipid changes. Reductions in LDL-cholesterol and triglycerides represent meaningful improvements in cardiovascular risk markers [50], but longer follow-up is needed to determine whether these changes persist and whether HDL-cholesterol continues to increase beyond the first postoperative year. Although not a primary outcome, 72% of participants were no longer receiving antihypertensive medications at 12 months, suggesting broader cardiometabolic improvement. However, medication discontinuation should be interpreted as a pragmatic indicator of de-escalation rather than formal remission, because standardized remission criteria for T2DM, dyslipidemia, and hypertension were not applied, and detailed information on medication dose changes, adherence, and prescribing decisions was not available.

The public healthcare setting further strengthens the relevance of these findings. Despite the effectiveness of bariatric surgery, access remains limited in Brazil: only approximately 10% of procedures are performed within the public health system, even though most of the population relies exclusively on public healthcare [20]. More recent eligibility-based estimates suggest even more restricted access, with only approximately 1% of potentially eligible adults without private health insurance undergoing bariatric surgery in the Brazilian public healthcare system from 2017 to 2023, with substantial regional disparities [21]. Real-world data from public reference centers are therefore important for describing outcomes under routine service conditions and supporting structured multidisciplinary bariatric care. In this context, our findings suggest that clinically meaningful short-term improvements in BMI and metabolic risk markers can be achieved in women treated within a standardized public bariatric surgery program. From a health-system perspective, these data support continued investment in structured multidisciplinary bariatric programs in the public sector, particularly for women with severe obesity and cardiometabolic comorbidities.

The open surgical approach also deserves consideration. Most contemporary bariatric literature focuses on laparoscopic RYGB, whereas the present cohort underwent open RYGB by laparotomy, reflecting institutional practice and surgical availability at the public reference center during the study period, when laparoscopic RYGB was not routinely performed. Different bariatric techniques may produce distinct anthropometric and metabolic trajectories because they differ in restrictive, malabsorptive, anatomical, and hormonal mechanisms. Thus, analyzing a single standardized procedure reduces heterogeneity and supports a more focused interpretation of postoperative metabolic changes. However, compared with laparoscopic access, laparotomy may be associated with greater postoperative pain, longer recovery, longer hospital stay, and a different perioperative complication profile, which could influence early mobility, dietary adaptation, follow-up adherence, and short-term BMI change. Thus, the early anthropometric trajectory observed in this cohort should be interpreted in light of the open surgical approach, even though the glycemic and lipid effects assessed here are primarily related to RYGB anatomy and physiology. Therefore, the laparotomy approach should be considered when comparing these findings with more recent laparoscopic series, but it does not diminish the relevance of the observed metabolic trajectories.

Although the present findings support substantial short-term anthropometric and metabolic improvement after RYGB, these benefits should be interpreted alongside the known risks and adverse effects of the procedure. RYGB may be associated with perioperative complications, marginal ulceration, dumping syndrome, internal hernia, hypoglycemia, and long-term micronutrient deficiencies, requiring careful patient selection and lifelong multidisciplinary follow-up [54,55,56,57]. Because postoperative complications and adverse effects were not systematically assessed in this retrospective cohort, the present results should be interpreted as describing metabolic effectiveness rather than the overall risk–benefit profile of the procedure.

This study has several strengths. The cohort was homogeneous, including only women who underwent the same surgical technique at a single public reference center. Surgical care and postoperative follow-up were provided by the same multidisciplinary team, reducing variability related to procedure type and care protocols. Multiple predefined postoperative assessments over 12 months allowed characterization of the timing of anthropometric, glycemic, and lipid changes, and longitudinal modeling accounted for repeated measurements within participants, strengthening analysis of temporal trends.

Some limitations should be acknowledged. The retrospective design and reliance on secondary data limited the availability of relevant variables, including blood pressure measurements, waist circumference, menopausal status, insulin-related markers, adherence to nutritional protocols, physical activity levels, and body composition measures. These unmeasured factors may have influenced the observed metabolic trajectories, as menopausal status and changes in fat mass, lean mass, and visceral adiposity can affect insulin resistance and lipid metabolism, while dietary adherence and physical activity may modify the magnitude and timing of postoperative BMI, glycemic, and lipid improvements. Excluding patients with incomplete follow-up may have introduced selection bias toward participants with better adherence to postoperative care and may therefore have overestimated the magnitude of postoperative improvement. Although complete-case analysis was considered appropriate given the limited attrition rate and the assumption that missingness was plausibly unrelated to the outcomes analyzed, this assumption cannot be confirmed in a retrospective cohort. The single-center design and women-only cohort improve internal consistency but limit generalizability and prevent direct comparison with men. The absence of a non-surgical control group means that observed changes cannot be attributed exclusively to surgery rather than concurrent dietary, behavioral, medication-related, or follow-up effects. The 12-month follow-up is appropriate for short-term evaluation but does not assess long-term durability, weight regain, or late HDL-cholesterol changes. Finally, medication discontinuation was analyzed descriptively and should not be interpreted as formal remission of T2DM, dyslipidemia, or hypertension.

## 5. Conclusions

Women with severe obesity undergoing RYGB at a public reference center experienced pronounced and clinically meaningful improvements in BMI, glycemic control, and lipid profile within 12 months. The observed trajectory was characterized by progressive BMI reduction, early stabilization of glycemic improvement, and more gradual reductions in LDL-cholesterol and triglycerides. These findings support the short-term effectiveness of standardized bariatric and multidisciplinary care in improving cardiometabolic risk markers in women, while longer follow-up is needed to assess durability, weight regain, and formal comorbidity remission.

## Figures and Tables

**Table 1:** Baseline demographic, clinical, anthropometric, and laboratory characteristics of the study population.

Characteristic	Overall Cohort (n = 150)
Age, years	43.69 ± 9.87
Ethnicity	
White	110 (73.3%)
Black	4 (2.7%)
Mixed race/Brown	31 (20.7%)
Other/not reported	5 (3.3%)
Place of residence	
Urban	138 (92%)
Rural	12 (8%)
BMI, kg/m^2^	45.4 ± 5.4
Baseline comorbidities	
Arterial hypertension	119 (79.3%)
Type 2 diabetes mellitus	57 (38%)
Dyslipidemia	46 (30.7%)
Current smoking	12 (8%)
Metabolic and laboratory markers	
Fasting plasma glucose, mg/dL	109 (103–117)
HbA1c	6.1 (6.02–6.3)
Total cholesterol, mg/dL	182.5 (175–188)
LDL-cholesterol, mg/dL	106.5 (101.5–111.5)
HDL-cholesterol, mg/dL	48 (46–50)
Triglycerides, mg/dL	143.5 (135–151)

Abbreviations: BMI, body mass index; HbA1c, glycated hemoglobin; LDL, low-density lipoprotein; HDL, high-density lipoprotein. Data are presented as mean ± standard deviation, median (interquartile range), or n (%), as appropriate.

**Table 2:** Longitudinal changes in anthropometric and metabolic parameters during the 12-month follow-up.

Time Point	Mean (95% CI)	Consecutive Comparison	Consecutive Difference	*p*-Value
BMI, kg/m^2^				
Preoperative	45.5 (44.5–46)			
30 days	40 (39.5–41)	Preoperative vs. 30 days	−5.5 (−6 to −4.5)	<0.001
3 months	36.5 (36–37.5) *	30 days vs. 3 months	−3.5 (−4.5 to −2.5)	<0.001
6 months	33.1 (32.5–34) *	3 months vs. 6 months	−3.5 (−4.4 to −2.5)	<0.001
12 months	31.5 (30.5–32) *	6 months vs. 12 months	−1.5 (−2.5 to −1)	<0.001
Fasting plasma glucose, mg/dL				
Preoperative	109 (103–117)			
30 days	93 (86–100)	Preoperative vs. 30 days	−20.5 (−31 to −9.5)	<0.001
3 months	93 (88.5–97.5) *	30 days vs. 3 months	0.5 (−11 to 11.5)	0.900
6 months	90 (86–94.5) *	3 months vs. 6 months	−3 (−11 to 4.5)	0.900
12 months	90 (84–95) *	6 months vs. 12 months	−0.5 (−9.5 to 8.5)	0.900
HbA1c, %				
Preoperative	6.10 (6.02–6.30)			
30 days	5.40 (5.17–5.62)	Preoperative vs. 30 days	−0.76 (−1.09 to −0.43)	<0.001
3 months	5.41 (5.26–5.56) *	30 days vs. 3 months	0.01 (−0.35 to 0.37)	1.000
6 months	5.40 (5.25–5.54) *	3 months vs. 6 months	−0.01 (−0.25 to 0.24)	1.000
12 months	5.36 (5.18–5.54) *	6 months vs. 12 months	−0.04 (−0.32 to 0.24)	1.000
Total cholesterol, mg/dL				
Preoperative	182.5 (175–188)			
30 days	163 (153–172.5)	Preoperative vs. 30 days	−19.5 (−33.5 to −5.5)	<0.001
3 months	161.5 (154.5–168) *	30 days vs. 3 months	−1.6 (−13.5 to 16.5)	0.900
6 months	161.5 (155–167.5) *	3 months vs. 6 months	0 (−10 to 10)	0.900
12 months	158 (150–165.5) *	6 months vs. 12 months	−3.5 (−15.5 to 8.45)	0.900
LDL-cholesterol, mg/dL				
Preoperative	106.5 (101.5–111.5)			
30 days	81.5 (45.5–117.5)	Preoperative vs. 30 days	−25 (−78 to 28)	0.700
3 months	88 (75–101)	30 days vs. 3 months	6.5 (−49 to 62.5)	0.900
6 months	89.5 (83.5–95.5) *	3 months vs. 6 months	2 (−19 to 22.5)	0.300
12 months	86.5 (79–94) *	6 months vs. 12 months	−3 (−17 to 11)	0.900
HDL-cholesterol, mg/dL				
Preoperative	48 (46–50)			
30 days	55 (43–67)	Preoperative vs. 30 days	7 (−11 to 25)	0.800
3 months	45 (40–50)	30 days vs. 3 months	−9.5 (−29 to 9.5)	0.900
6 months	51 (49–53.5)	3 months vs. 6 months	6 (−2 to 13.5)	0.300
12 months	55 (52–58)	6 months vs. 12 months	4 (−2 to 10)	0.300
Triglycerides, mg/dL				
Preoperative	143.5 (135–151)			
30 days	116.5 (59.5–174)	Preoperative vs. 30 days	−26.5 (−110 to 57.5)	0.900
3 months	118 (98–138)	30 days vs. 3 months	1.5 (−87 to 90)	0.900
6 months	95.5 (86–105) *	3 months vs. 6 months	−23 (−55 to 9.5)	0.900
12 months	90.5 (78–103) *	6 months vs. 12 months	−5 (−22 to 25.5)	0.900

Data are presented as estimated mean (95% confidence interval). Time points correspond to the preoperative assessment, 30 days, 3 months, 6 months, and 12 months after open Roux-en-Y gastric bypass. Asterisks indicate values significantly different from the preoperative assessment in post hoc comparisons. Asterisks indicate values significantly different from the preoperative assessment in post hoc comparisons. 95% CI, 95% confidence interval; BMI, body mass index; HbA1c, glycated hemoglobin; LDL, low-density lipoprotein; HDL, high-density lipoprotein. * Significantly different from the preoperative value for the corresponding variable, *p* < 0.01.

## Data Availability

The data supporting the findings of this study are available from the corresponding author upon reasonable request.
